# Social Networks Shape the Transmission Dynamics of Hepatitis C Virus

**DOI:** 10.1371/journal.pone.0011170

**Published:** 2010-06-23

**Authors:** Camila Malta Romano, Isabel M. V. Guedes de Carvalho-Mello, Leda F. Jamal, Fernando Lucas de Melo, Atila Iamarino, Marco Motoki, João Renato Rebello Pinho, Edward C. Holmes, Paolo Marinho de Andrade Zanotto, the VGDN Consortium

**Affiliations:** 1 Laboratory of Molecular Evolution and Bioinformatics, Department of Microbiology, Biomedical Sciences Institute – ICBII, University of São Paulo, São Paulo, Brazil; 2 Viral Immunology Laboratory, Butantan Institute, São Paulo, Brazil; 3 Laboratory of Tropical Gastroenterology and Hepatology, Department of Gastroenterology, Institute of Tropical Medicine, School of Medicine, University of São Paulo, São Paulo, Brazil; 4 Training and Reference Center DST/AIDS, Control Diseases Center, Secretary of Health of São Paulo State, São Paulo, Brazil; 5 Mueller Laboratory, Department of Biology, Center for Infectious Disease Dynamics, The Pennsylvania State University, University Park, Pennsylvania, United States of America; 6 Fogarty International Center, National Institutes of Health, Bethesda, Maryland, United States of America; Institut Pasteur, France

## Abstract

Hepatitis C virus (HCV) infects 170 million people worldwide, and is a major public health problem in Brazil, where over 1% of the population may be infected and where multiple viral genotypes co-circulate. Chronically infected individuals are both the source of transmission to others and are at risk for HCV-related diseases, such as liver cancer and cirrhosis. Before the adoption of anti-HCV control measures in blood banks, this virus was mainly transmitted via blood transfusion. Today, needle sharing among injecting drug users is the most common form of HCV transmission. Of particular importance is that HCV prevalence is growing in non-risk groups. Since there is no vaccine against HCV, it is important to determine the factors that control viral transmission in order to develop more efficient control measures. However, despite the health costs associated with HCV, the factors that determine the spread of virus at the epidemiological scale are often poorly understood. Here, we sequenced partial NS5b gene sequences sampled from blood samples collected from 591 patients in São Paulo state, Brazil. We show that different viral genotypes entered São Paulo at different times, grew at different rates, and are associated with different age groups and risk behaviors. In particular, subtype 1b is older and grew more slowly than subtypes 1a and 3a, and is associated with multiple age classes. In contrast, subtypes 1a and 3b are associated with younger people infected more recently, possibly with higher rates of sexual transmission. The transmission dynamics of HCV in São Paulo therefore vary by subtype and are determined by a combination of age, risk exposure and underlying social network. We conclude that social factors may play a key role in determining the rate and pattern of HCV spread, and should influence future intervention policies.

## Introduction

Hepatitis C virus (HCV) is a major cause of hepatitis and liver cancer globally, chronically infecting nearly 3% of the world's population [1]. During the second half of the 20^th^ Century, the global HCV pandemic worsened due to widespread viral transmission by blood or blood-derived products, and by unsafe medical practices. Today, needle sharing among injecting drug users (IDUs) is a major risk factor in industrialized countries [2] and prevalence among IDUs ranges from 30 to 90% [3], [4], with between 5.6 to 36% of Brazilian drug users infected [5]. Approximately 15–40% of infected persons clear the virus during the acute phase. Of the remainder, nearly 80% result in chronic infections which may lead to subsequent viral transmission [6]. It has been estimated that 0.8% to 3.5% of the Brazilian population is HCV-positive, while prevalence among those that are also HIV infected may reach 25% [7].

HCV is classified into six major genotypes and many subtypes, varying in geographical distribution, transmission route and treatment response [1], [8], [9]. Genotype 1, and especially subtypes 1a and 1b, are the most prevalent worldwide, although 1b is mostly found among older members of the population who have a history of blood transfusion [3], [10], [11]. HCV-1a and 3a appear to have emerged recently and are spreading rapidly in many global regions, with a high prevalence among young IDUs [3], [10], [11], [12]. Coarse-grained phylogenetic studies suggest that HCV subtypes 1a and 1b reached a plateau globally in the 1980's after previously experiencing exponential growth [13]. Despite the increasing importance of HCV for human health, no vaccine is currently available, treatment is suboptimal in many countries and, alarmingly, up to 40% of the transmission events are of unknown cause [14]. Moreover, there is a severe lack of donor organs, causing tens of thousands of patients with decompensate liver disease and portal hypertension to await orthotopic liver transplantation [15].

The lack of an effective vaccine highlights that prevention is currently the main strategy available to control viral transmission. As a consequence, detailed studies of the molecular epidemiology of HCV, particularly at the scale of local epidemics, are needed to enhance our understanding of the transmission dynamics of this major public health problem. Herein, we investigated the population dynamics of HCV in the most populous region of Brazil – São Paulo state – with the aim of identifying the epidemiological factors that influence viral transmission.

## Materials and Methods

### Experimental design

Partial NS5b gene sequences were obtained from blood samples collected from 591 patients chosen irrespective of their risk associations between 1997 and 2006 as part of the Viral Genetic Diversity Network (VGDN) program [16]. Patients were sampled from four different regions within São Paulo state; Ribeirão Preto, São José do Rio Preto, São Bernardo do Campo and from two reference centers for HCV surveillance and treatment in São Paulo city (Clínicas Hospital and Reference and Training Center- RTC). After RNA extraction from blood samples and cDNA synthesis, a 361 nt region of NS5b was amplified. Questionnaire data from patients such as age, gender, sex, and marital state were collected at the time of sampling. Relevant clinical information on transmission risk factors was gathered, including: (*i*) self-declared drug use profile: injected (IDU) or non-injected drugs (crack and cocaine) (NIDU), (*ii*) sexual behavior, including the number of casual and regular sexual partners during their entire life and, (*iii*) blood transfusion (BT) before and after 1993 when HCV blood screening started in Brazil, were also obtained. The NS5b sequences were typed by phylogenetic inference with the inclusion of worldwide HCV reference sequences available in GenBank (http://www.ncbi.nlm.nih.gov/Genbank/) and Los Alamos HCV database (hcv.lanl.gov/). All sequences generated here have been deposited in GenBank and assigned accession numbers GQ490493–GQ491027.

### Ethics Statements

All procedures adopted in this work were done according to the terms agreed by the Ethics Committee on Human Research of the University of São Paulo and informed consent terms were signed by all patients.

### Inferring phylogenetic history

NS5b sequences were aligned using Clustal X [17]. Phylogenetic trees were inferred using the GARLi program (Genetic Algorithm for Rapid Likelihood Inference) [18], which employs an extensive branch-swapping protocol and optimizes the substitution model iteratively during the search. Initial trees were used to determine the position of our sequences in the context of the global HCV pandemic. In particular, these analyses allowed us to identify and eliminate sequences that were directly related to non-Brazilian sequences suggesting that they had been imported from localities outside of Brazil.

### HCV phylodynamics

To investigate the population history of HCV in São Paulo state (after excluding potential migrants) we utilized the Bayesian Markov Chain Monte Carlo (MCMC) approach implemented in the BEAST package [19]. First, the rate of nucleotide substitution as well as the Time to the Most Recent Common Ancestor (TMRCA) of each viral subtype was estimated under the best-fit model of nucleotide substitution obtained by comparing the GTR+Γ+I, GTR+Γ+I+SRD_112_, HKY and HKY+SRD_112_ models [20] using Bayes factors (BF) (although similar results were obtained under all substitution models). We also utilized both strict and relaxed (uncorrelated lognormal) molecular clocks. To be as conservative as possible we also employed the least constrained Bayesian skyline coalescent prior, which provided similar substitution rates to those obtained with the best demographic model for each subtype. To improve the MCMC search, we set the operator values as a function of the number of taxa in each group, and set the upper value of the ‘population size’ parameter to 170 million, which hypothetically covers the estimated number of infected people in the world. After optimizing the values of the MCMC operators during preliminary runs, up to 10 additional MCMC runs, each consisting of 20 million generations (with a 50% burnin), were undertaken to obtain convergence of parameter estimates. With the substitution rates in place we then estimated the rate of population growth (*r*) under the demographic model (i.e. constant, exponential population growth or logistic population growth) that best-fit each subtype data set, with model comparisons again undertaken using BF. Similarly, setting the prior on the substitution rate, we estimated Bayesian skyline plots for each data set. In all cases the convergence of parameters during the MCMC was inspected with Tracer v.1.4 [19], with uncertainties depicted as 95% highest probability (HPD) intervals. To provide an independent assessment of the robustness of our analysis of evolutionary dynamics we also estimated the TMRCA of each data set using the Path-O-Gen program (available at http://tree.bio.ed.ac.uk/software/pathogen/), which employs a linear regression of root-to-tip genetic distances against sampling time based on the ML phylogenies described above.

### HCV phylogeography

To evaluate how phylogenies are shaped by underlying geography within São Paulo state we utilized the Bayesian approach available in BEAST [19]. Specifically, we performed a phylogeographic analysis using a standard continuous-time Markov chain (CTMC) model with the Bayesian stochastic search variable selection (BSSVS) procedure [21]. The three main geographic locations of sampling (Metropolitan area of São Paulo, Ribeirão Preto and São José do Rio Preto) were coded as multistate characters and the most parsimonious description of geographic spread was obtained by MCMC sampling from the plausible set of trees using the procedures outlined above.

### Fitting a power law to the sexual contact data

We estimated the extent of social interaction among the patients in our data set as their total number of sexual contacts [22]. We fitted a pure power law distribution model of the form *P*(*k*) ≅ *k* ^−α^ to our data and estimated the minimal value of sexual contacts above which the data follow a power law (*x*_min_), and the maximum likelihood estimator of the scaling exponent α using the goodness-of-fit based method of [23] with the R v.2.10.0 program (http://www.r-project.org/). The quality of fit was evaluated by a Kolmogorov-Smirnov goodness-of-fit (*K–S*) test that compares the *D*-statistic for the observed data to a critical *D* value estimated for large *n* [24] from the expected power law distribution.

### Estimating *R*

To allow a broad-scale comparison of growth dynamics across HCV epidemics we obtained approximate estimates of the basic reproductive number (*R*) for each HCV genotype using the relation *R* = 1+*rD*, where *r* is the population growth rate and *D* is the mean time of infectiousness [25]. Growth rate (*r*) estimates were obtained with BEAST from the best-fit demographic model for each data set. Additionally, we estimated *r* using GENIE (http://evolve.zoo.ox.ac.uk/software/Genie/) under the relation *r* = ρµ, where µ is the nucleotide substitution rate (subs/site/year) and the composite parameter ρ (*r*/µ) estimated from the ML phylogeny, with branch lengths adjusted for date of sampling with the TipDate program [26]. Parameter *D* is an estimator of the generation time distribution (*w*(*t*)), which is a function that describes the distribution of times between infections in a chain of transmission [27]. Accurate proposals for *w*(*t*) depend on several complex factors that are difficult to evaluate for a persistent virus such as HCV, which causes fluctuating viral load levels during chronic infections that may last several years [28], [29]. Moreover, the probability of virus transmission is expected to vary according by risk factor. Therefore, instead of utilizing *w*(*t*), *R* was calculated using a mean time of infectiousness (*D*) of 2, 5, 10, and 15 years, which appear to cover a reasonable range of values. Importantly, values of *R* inferred from phylogenetic trees may not relate linearly to the number of infected people, and may also be strongly affected by the variance on the number of potential infectious contacts events. These limitations notwithstanding, agreements between *R* estimated from viral phylogenies and from epidemiological data have been noted [30].

Finally, the human population growth rate was approximated using census data in time fitted to both exponential and logistic curves, and by estimating the rate coefficients under the model that best fit the data using the SPSS v.11.0.1 program (SPSS Inc. Chicago, IL).

## Results

### HCV epidemiology and phylogeography

A total of 591 HCV sequences obtained from São Paulo State were successfully amplified, sequenced and typed by phylogenetic analysis using reference sequences obtained from the Los Alamos HCV database. Genotype 1 was the most prevalent (70.5%), comprising 51.3% of subtype 1b and 48.7% of HCV-1a samples. Subtype 3a was the next most prevalent, representing 24.2% of all samples. Genotype 2 included subtypes 2b and 2c and was found at frequencies of 2.1% and 1.9% of the population, respectively. Genotypes 4 and 5, not commonly found in Brazil, were detected in five patients, one infected with the subtype 4a and four with 5a. Subtypes only found in a small number of patients (2, 4 and 5) were discarded for the subsequent analysis. Phylogenies of the most common 1a, 1b and 3a subtypes also suggested different demographic histories (Figure S1). Specifically, the HCV-1b phylogeny possessed three distinct clusters comprising Brazilian sequences, such that the same local epidemic is associated with different 1b lineages. In contrast, there was no evidence for phylogenetically distinct clusters in trees of subtypes 1a and 3a.

**Figure 1:**
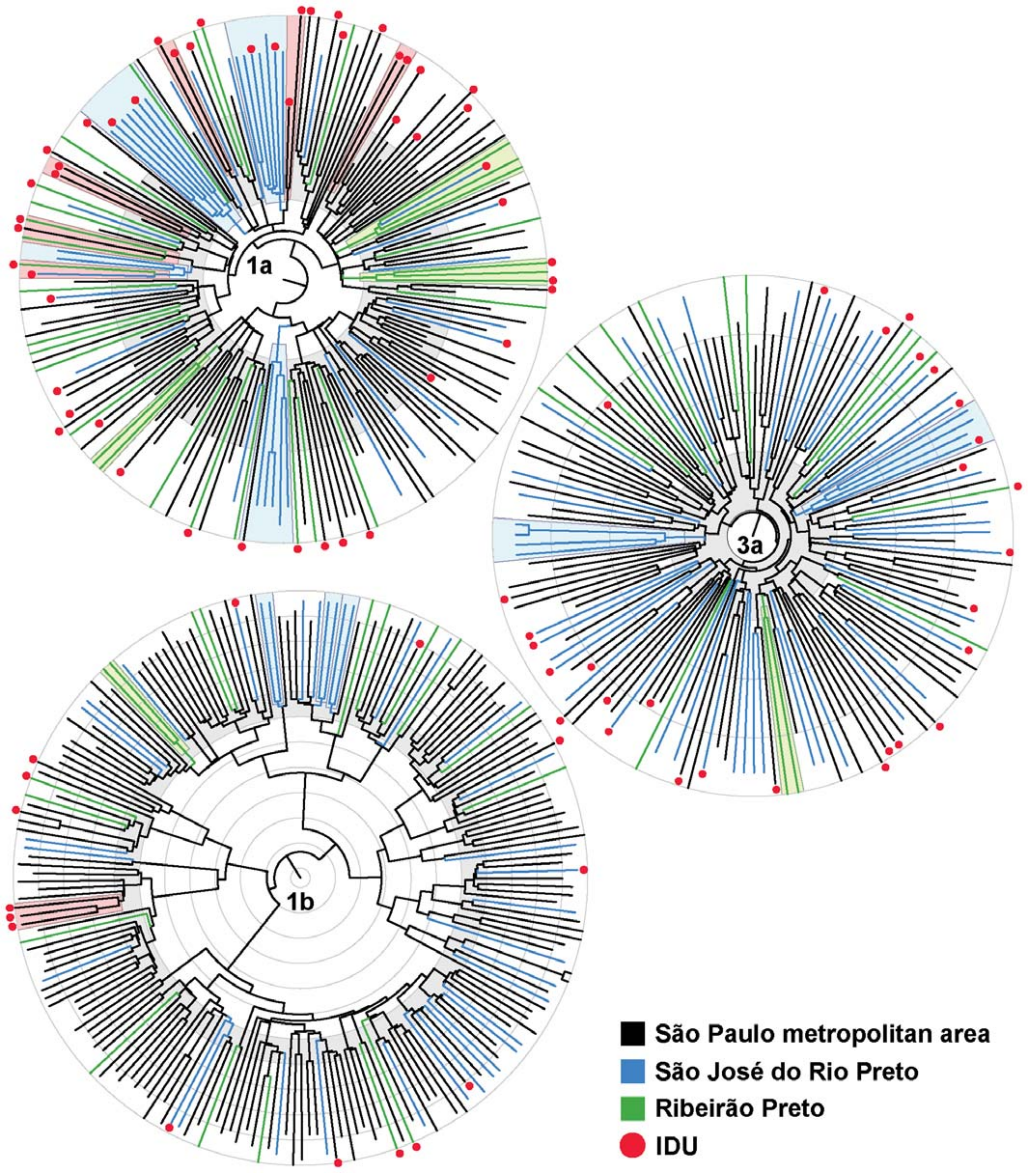
Phylogeographic and IDU risk analysis. We show Maximum Clade Credibility (MCC) trees for the main HCV genotypes in São Paulo. Branches are colored according to the geographic locality of sampling, with IDUs shown as red dots at the tips. The highest posterior probability for the root of all trees revealed that the epicenter of HCV epidemics was the metropolitan area of São Paulo. Highlighted sectors indicate ancestral nodes with posterior probability >50% located outside the metropolitan area of São Paulo (green in Ribeirão Preto and blue in São José do Rio Preto). Branch lengths are scaled in years shown as concentric rings of 5 years. The grey concentric rings indicate that the epidemic expansion of HCV-1a in São José do Rio Preto and Ribeirão Preto was synchronized with the growth in the metropolitan area of São Paulo. Most of the IDUs are located within the HCV-1a and HCV-3a subtypes. Sectors highlighted in red indicate IDU clusters with posterior probability >50%. All trees depicted non-IDU as the root state with the highest posterior probability.

To reduce the effect of any phylogeographic structure on our subsequent analysis of transmission dynamics we first excluded all taxa that we suspected to be “migrant” due to their close phylogenetic relationship to samples obtained from outside Brazil (and taken from GenBank). In addition, we performed a Bayesian analysis of phylogeographic structure, dividing our time-stamped sequences into three main geographic sources: (*i*) the Metropolitan area of São Paulo city, including patients from the Clinicas Hospital, the Reference and Training Center (RTC), and São Bernardo dos Campos (located in the outskirts of São Paulo), (*ii*) Ribeirão Preto, located in the west of São Paulo state some 317 km from the São Paulo metropolis, and, (*iii*) São José do Rio Preto in northern São Paulo state, 443 km from the São Paulo city and 205 km from Ribeirão Preto. For each HCV genotype, the root location with the highest posterior probability falls in the metropolitan area of São Paulo city and is shown as black branches in the maximum clade credibility (MCC) trees (Figure 1). Although we found small phylogenetically distinct clusters of sequences spreading in Ribeirão Preto (sectors highlighted in green) and São José do Rio Preto (highlighted in blue) in all three trees, overall there was clearly no segregation by region of sampling. In addition, all region-specific clusters radiated at approximately the same time as those from São Paulo (grey concentric circles in Figure 1) suggesting that they should not impact significantly on overall dynamics.

### Time-scale of HCV emergence and dynamics in São Paulo

**Figure 2:**
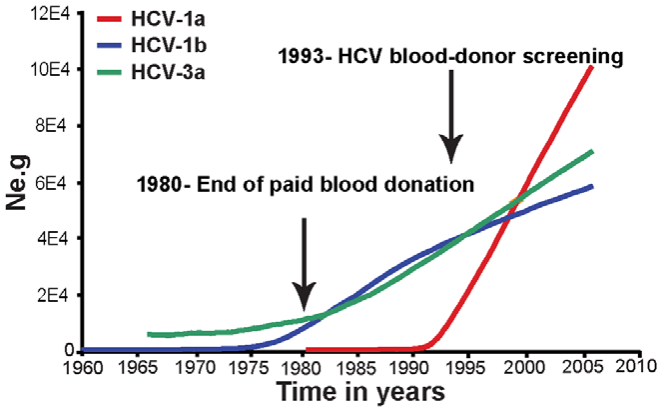
Patterns of population growth for HCV1a, 1b and 3a. The colored lines in the strict clock Bayesian skyline plots show the superimposed median values (*y*-axis) of relative genetic diversity (*Ne.g*, where *Ne* is the effective population size and *g* the inter-host generation time) through time in years for each subtype. Vertical arrows indicate the introduction of control measures in 1980 (end of paid donation) and 1993 (implementation of screening for HCV in blood banks) aimed to hamper HCV spread by blood products in Brazil.

Key demographic and epidemiological parameters estimated for all HCV subtypes, or collected from patients' questionnaires, are shown in Table 1. Importantly, our estimates for the TMRCA of each subtype obtained under the best-fit demographic model covered essentially the same range as those estimated by the Bayesian skyline analysis, suggesting that these results are robust to demographic history (see supplementary Table S1A to S1F for details of the statistical analysis). Although HCV subtypes 1a, 1b and 3a showed similar rates of nucleotide substitution, there were significant differences among subtypes in both TMRCA and population growth rate (Table 1). To infer how the demographic history of each subtype changed through time we inferred Bayesian skyline plots using a relaxed molecular clock and the substitution rates estimated previously under the best-fitted model of nucleotide substitution (that is, GTR+Γ+I+SRD_112_ for HCV-1a, and 1b and HKY+SRD_112_ for HCV-3a). Although the 95% HPD values for the skyline plots under the relaxed clock were too wide for meaningful interpretation, those generated using a strict clock gave a reasonable demographic signal (Figure 2 and S2).

**Table 1:** Risk factor, demographic and epidemiologic parameters of HCV.

									*Evolutionary*	*dynamics*			
HCV	Taxa		*Risk*	*factors*	*(%)*		Substitution rate	TMRCA	*r*		*R* (BEAST/	GENIE)	
Subtype	(#)	BT	IDUs	Tattoo	NIDU	Unknown	(95% HPD)	(95% HPD)	(95% HPD)	2	5	10	15
1a	183	25.1	26.9	28	18.3	27.9	1.9×10^−3^	1981	0.53	2.06	3.65	6.3	8.95
							(7.5×10^−4^ 3×10^−3^)	(1958–1992)	(0.38–0.67)	(2.4)	(4.4)	(7.8)	(11.3)
1b	205	47	7.5	10.6	8.5	37.6	9.2×10^−4^	1927	0.24	1.4	2.2	3.4	4.6
							(1.4×10^−4^–1.8×10^−3^)	(1740–1981)	(0.17–0.36)	(1.2)	(1.6)	(2.2)	(2.8)
3a	147	36	20	17	12.1	30.6	1.2×10^−3^	1966	0.40	1.8	3.0	5.0	7.0
							(2.5×10^−4^ –2.4×10^−3^)	(1888–1999)	(0.29–0.49)	(1.6)	(2.6)	(4.2)	(5.8)

Growth rate (*r*) is the median values estimated by the growth model (exponential for HCV-1a and 3a and logistic for HCV-1b) that best-fit the data. Nucleotide substitution rates per site, per year and TMRCAs were estimated in Bayesian skyline runs using a relaxed uncorrelated lognormal molecular clock. The *R* values were estimated from data generated by BEAST and GENIE (in parenthesis) for mean duration of infectiousness (*D*) values of 2, 5, 10 and 15 years. BT = blood transfusion; IDU = injecting drug user; NIDU = non-injection drug use; unknown = patients with no history of being arrested, blood transfusion, tattoo, injecting or non-injecting drug use, HIV negative and no HCV positive partners.

Together, these evolutionary analyses suggest that HCV-1b was the first subtype to enter São Paulo and which experienced rapid population growth in the 1970's, possibly spread by blood transfusion and unsafe medical practices (the frequency of each risk factor is given in Table 1). HCV-1b was followed by HCV-3a in the mid 1960s (Table 1). HCV-1a was most likely the last subtype to emerge and started growing exponentially in 1990's, after the end of paid blood donation and the onset of obligate blood-donor screening (Figure 2), attaining the highest growth rate of all (Table 1). Subtypes HCV-1a and 3a are known to dominate IDU-associated infections in most industrialized countries. However, we found only 27% of HCV-1a and 20% of HCV-3a infected individuals in our study to be at risk of exposure by IDU, and a similar proportion, albeit with lower numbers, of NIDU (Table 1). This last result is of relevance since people that use non-injecting drugs (NIDU) are also at risk of HCV infection, mainly due to crack and cocaine [31]. Indeed, approximately half of our NIDU patients that reported sharing cocaine paraphernalia also reported nasal bleeding (76 patients). Interestingly, most of our NIDU used cocaine exclusively, with a small percentage (6%) also reporting smoking crack, indicating that crack may not be particularly important for HCV transmission among our study population. Finally, because HCV prevalence in IDUs can reach 90% [32], it is possible that IDUs act as reservoirs that disseminate the virus through the population as a whole.

We therefore explored the impact of injecting drug use on the HCV transmission network. To do this we reconstructed the ancestral mode of transmission across our subtype phylogenies using “IDU”, “non-IDU”, and “unknown status” as character states. This revealed a non-IDU root in all cases, suggesting that HCV transmission did not originate exclusively from IDU (Figure 1). However, we did observe six small IDU clusters in the HCV-1a phylogeny and one cluster in the HCV-1b tree (highlighted red sectors in Figure 1). Moreover, since IDU occurrence was higher in HCV-1a than HCV-3a and HCV-1b, we also tested if IDU could explain the differences in growth rates we observed. By excluding the IDUs from HCV-1a data set, we observed that the exponential growth rate only increased from 0.51 to 0.56 and with overlapping 95% HPDs. We also observed that 28 to 38% of our patients had unknown risk factors for HCV transmission, which suggested that additional host-associated factors should be considered as determinants of transmission dynamics.

### Dynamics associate with age structure of HCV patients

**Figure 3:**
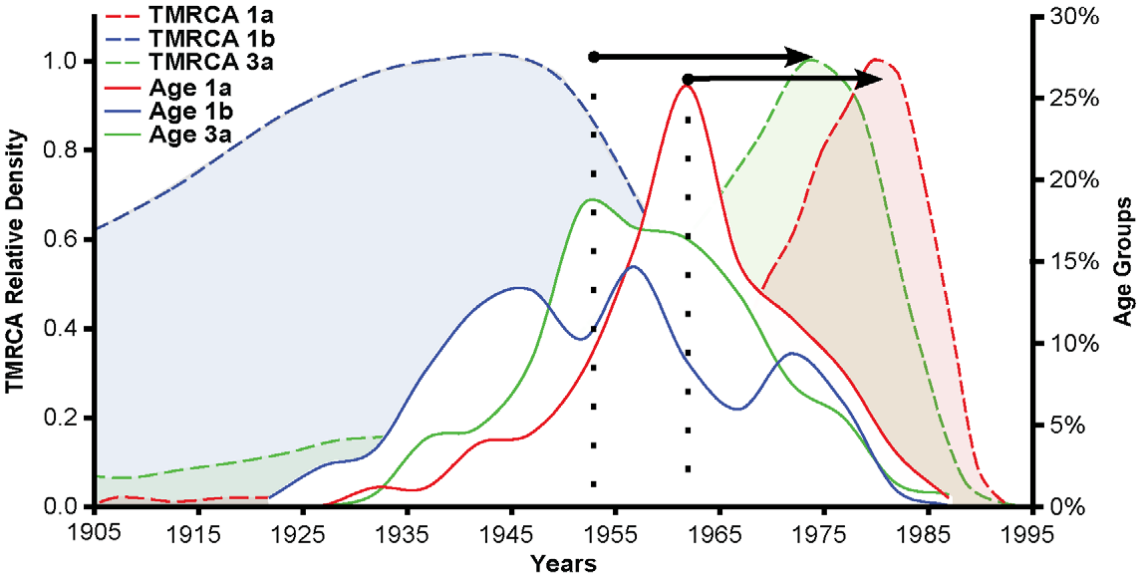
Viral and host age structure. Dotted color-filled distributions at the back of the figure show the relative densities of estimates of the Time to the Most Recent Common Ancestor (TMRCA) of HCV-1a (red), HCV-1b (blue) and HCV-3a (green). The TMRCA relative densities (left *y*-axis) depict the frequency normalized to unity of TMRCA values sampled during the MCMC runs in BEAST. Full line distributions in the foreground show the percentage of HCV carriers that were born in a specific year (*x*-axis). Patient age distributions were color-coded in the same fashion as the TMRCA. Horizontal arrows, connecting the mode of age of birth of patients with the TMRCA peak value for both HCV-3a and HCV-1a, show the temporal correlation between each subtype origin of spread and the age of its carriers.

An important observation from our analysis of HCV population dynamics was that viruses that emerged more recently – subtypes 1a and 3a –also experienced higher rates of population growth. Strikingly, the year of birth of people infected by HCV-1a and 3a was uni-modal, with modes of around 1954 and 1964 for HCV-3a and HCV-1a, respectively (Figure 3). In marked contrast, the year of birth structure of HCV-1b carriers was clearly multi-modal, with three peaks roughly spaced according to decade-sized intervals. This tri-modal distribution may be explained by the fact that subtype HCV-1b has likely been circulating in São Paulo since the beginning of the 20^th^ century, and because carriers survive for long time periods following infection. Therefore, people infected several decades ago were sampled during our study.

### Host population size and social networks impact on HCV transmission

The population dynamics of infectious agents are greatly influenced by a variety of epidemiological factors, including host population size and density, the spatial distribution of the host, and the rate of contact between individuals [33], [34]. As an initial exploration of how these factors may impact HCV transmission we investigated census population growth concurrent with the spread of HCV in São Paulo. This analysis revealed a positive relationship between human population increase and the growth rates of each HCV subtype according to their time of origin (Figure S3).

**Figure 4:**
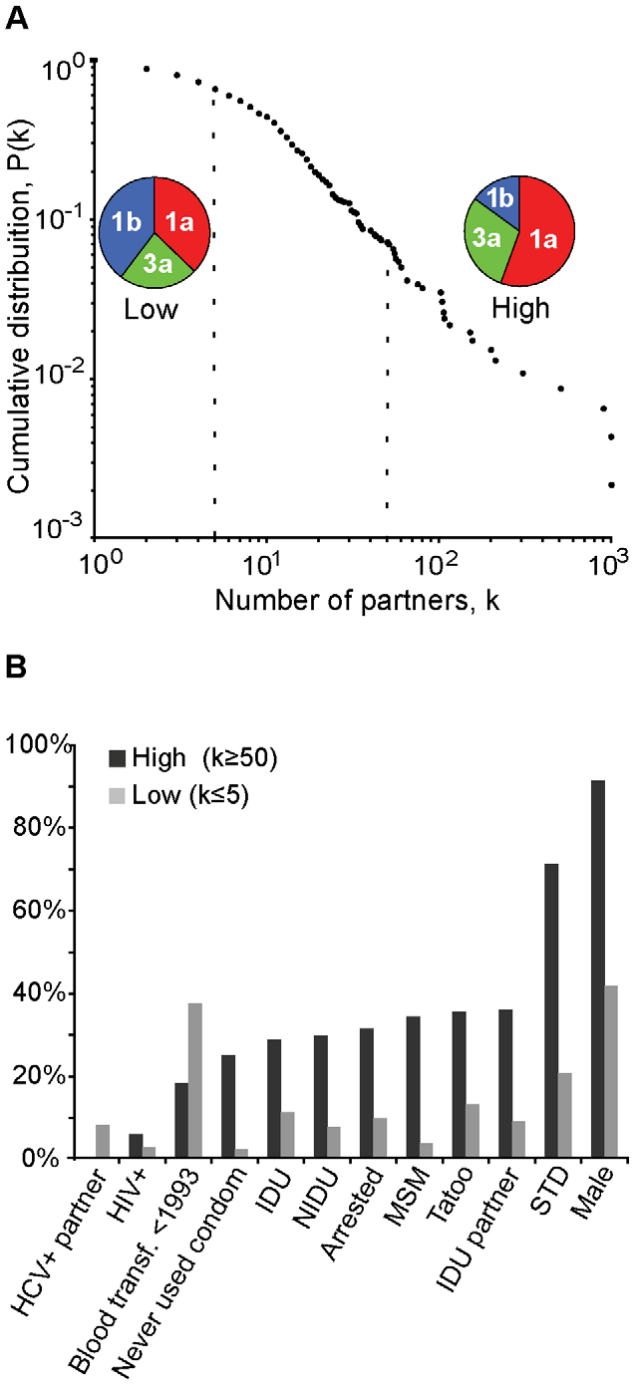
Scale-free sexual contact network of HCV carriers. **A**) The cumulative frequency distribution *P*(*k*) versus the number of partners (*k*) shows that the low connectivity end of this network (individuals with 5 or less sexual partners) is associated with a higher prevalence of HIV-1b infected individuals compared to the high connectivity heavy tail-end of the network, which is in turn more frequently associated by HCV-1a and 3a carriers. **B**) Social, clinical and transmission risk factors are shown in increasing frequency in relation to the high-connectivity group that includes all HCV patients with 50 or more sexual contacts (dark grey) compared to the low-connectivity group that includes all HCV patients with 5 or less sexual contacts (light grey). The final two columns on the right show a preponderance of males with increased risk factors and behaviors. The high frequency of blood transfusions among the low-connectivity group shows its importance for HCV transmission among the general population at large up until the early 1990's.

Since the rate of contact between infected and susceptible hosts (*i.e.*, the nodes of a transmission network) are crucial determinants of epidemic dynamics [35], [36], the number of connections per highly-connected node should increase as a function of host population density and size. Moreover, it reflects the intensification of social interaction as measured by the number of sexual contacts [22]. We therefore examined the dependency of the cumulative frequency distribution *P*(*k*) on the total number of sexual partners (*k*) obtained from questionnaires available for 492 of the 591 patients in our study group. Strikingly, we observed a negative relationship of *P*(*k*) for increasingly higher values of the number of reported sexual contacts (*k*), showing stereotypically sequential decay on the number of sexual contacts (Figure 4A). We then fitted the data to a pure power law distribution and estimated the value of x_min_ = 10 and the maximum likelihood value of the scaling exponent α = 2.15. Although other heavy-tailed distributions may explain our data [23], it conformed well to a power law as shown by the significance of the *K–S* test statistic *D* = 0.0254 (p<0.001, *n* = 217, *D-critical*≅0.0913), expounding the scale-free nature of this HCV network. In general, values of *k* for most of the cumulative frequency values of *P*(*k*) indicated a higher number of sexual contacts and connectivity for HCV-1a, followed by HCV-3a and finally HCV-1b, and most strongly so at the heavy-tail side of the curves (Figure 4A).

Since our patients did not identify sexual partners among others in the cohort, we were not able to infer the exact topology of the HCV network. Nevertheless, we examined the social, risk-related and clinical factors at the two ends of the overall distribution (*i.e.*, for all HCV carriers taken together to obtain robust parameter estimates). By dividing all patients into two extreme log-binned groups with “low-connectivity”, defined as 5 or less sexual partners (40% HCV-1b, 37% 1a and 23% 3a), and “high-connectivity”, reflecting 50 or more sexually partners (56% HCV-1a, 30% 3a and 14% 1b), we found that greater sexual activity was also associated with increased exposure to known HCV risk factors (STD, drug use, tattooing and imprisonment) and specific behaviors, such as unprotected sex (Figure 4B).

### HIV-1 and HBV co-infection

HCV, HIV-1, and hepatitis B virus (HBV) share similar routes of transmission and HCV infects 25 to 30% of HIV-infected persons [37]. Although our sampling protocol was designed to minimize the inclusion of persistent viral co-infections, we in fact sampled 36 HIV-1 positive out of 878 HCV carriers. This 4.1% prevalence was six times higher than the HIV-1 prevalence of 0.7% in the general Brazilian population [www.ias-2005.org/admin/images/upload/533.pdf]. From the seventeen co-infected samples sequenced here, there were nine patients infected by HCV-1a, four by 1b and four by 3a. From these, 2.7% where at the low-connectivity end of the HCV network and 6.1% were located at the high-connectivity end, which also had several other evident risk factors associated other than HIV-1 (Figure 4B). Therefore, some small proportion of the increase in growth rate we see in 1a and 3a compared to 1b could have been due to an increased rate of HCV transmission due to HIV co-infection (Figure 4B). Similarly, we found two HCV patients from a total of 494 to be HBV positive (0.4% prevalence, which was 10 times lower than prevalence of 3.7% in metropolitan areas of Brazil [38]. Both these individuals had medium connectivity (from 6 to 49 sexual partners) in the HCV network.

## Discussion

Our study reveals that co-existing yet distinct epidemics of different HCV subtypes circulate in São Paulo and among discrete age-structured groups that are differently exposed to risk factors for viral transmission. Subtypes HCV-1a and 3a also grew faster than 1b during the last 20 years, and not reaching the plateau observed in other localities [13]. Since our results are based on the analysis of a large data set of randomly sampled sequences from a one single regional epidemic, and backed by data on risk factors and exposure behaviors, we believe that they are potentially a more accurate reflection of HCV epidemic dynamics. We found that the metropolitan area of São Paulo city was the epicenter of HCV spread in the State, coinciding with it being also the epicenter of human population growth during the 20th century. Perhaps because HCV-1b reached high prevalence earlier in the 20th century it has been able to establish sustained transmission among generations born from the 1940's to the 1970's. In addition, subtype 1b was mainly transmitted through contaminated blood transfusion, which is not obviously associated with an age-specific risk group, and the range of patients ages is expected to be large in this case. This is reflected in the fact that 47% of HCV-1b carriers born in the 1970's (*i.e.*, members of the last peak) received blood transfusion at an early age. In contrast, HCV-3a and subsequently HCV-1a colonized increasingly larger waves of susceptible hosts. These results agree with previous studies of HCV epidemics using smaller sample sizes showing that HCV-1a has grown faster than 1b in many regions, including Brazil [12], [39], [40], [41], [42]. While the nucleotide substitution rate we obtained for HCV-1a (Table 1) was slightly higher than those estimated for HCV in other localities [12], it is possible that this signifies an increased strength of natural selection in this case, in turn reflecting an elevated epidemic growth rate. Indeed, tree-based estimates of the basic reproductive number (*R*) for the HCV-1a and HCV-3a epidemics in São Paulo were higher than those observed among IDUs in the United Kingdom [12], which also exhibits a lower rate of census population growth during the last century (see Figure S4 A and C). However, because there was no clear distinction in risk association between subtypes HCV-3a and HCV-1a, and because their growth rates were associated with initial time of spread, it is clear that additional factors are needed to fully explain the differing population dynamics of these subtypes.

We therefore propose that the association between age modes and times of subtype origin (horizontal arrows in Figure 3) is largely explained by social factors that might confine HCV transmission within specific age groups. Both an age group restriction on transmission and the control measures implemented in Brazil in 1980 and 1993 could help explain the slowdown in HCV-1b transmission that occurred after 1985 (Figure 2). In addition, age structure may explain more than just the epidemics in São Paulo since the lower number of young adults (between 20 to 39 years of age) in the UK compared with São Paulo is compatible with their difference in viral growth rates (Figure S4 B). It is likely that age-structured waves of HCV carriers transmit the virus among age-related susceptible hosts, perhaps due to behavioral generation exclusion (*i.e.* a “generation gap”). This accords with the observation that people generally only have significant contact with others like themselves (“the homophylic principle”), which governs the emergence of assortative mixing in social networks [43]. When assortative mixing occurs among highly connected individuals (which can be thought of as “hubs”), such that nodes with many connections tend to be connected to other nodes with many connections (*i.e.* preferential attachment), then scale-free networks emerge such as the one we document here [44], [45]. Although blood transfusion and IDU enhance HCV transmission, it has been also shown that “survival” sex work among young people was the strongest predictor of elevated HCV incidence in Canada [46]. Importantly, this justifies our sexual-contact network analysis and also agrees with our results on age stratification (Figure 3) and risk factors among highly connected patients (Figure 4).

Importantly, although we do not currently understand to which extent HCV is sexually transmitted, the association of high connectivity and risk behavior in the HCV network, agrees with genotype composition and age structuring, since 29% of the low-connectivity patients were older than the high-connectivity group. This also fits with the observed trend of higher rates of viral population growth among younger age groups. The exception to this was the obviously increased risk of blood transfusion among the low-connectivity group (with a particularly high prevalence in HCV-1b carriers), which also had a more even sex ratio compared to the male-dominated high-connectivity end of the spectrum. Moreover, both extreme groups had marked differences in other factors, such as STD, IDU partners, tattooing, MSM, imprisonment, IDU, NIDU, not using condoms, etc., which could impact on the rates of contact for HCV transmission (Figure 4B). These data, together with the age-stratification data shown in Figure 3, are strongly suggestive of assortative mixing.

In sum, the patterns obtained from clinical and sociologic data suggest that each HCV subtype analyzed here is characterized by different social networks. We therefore propose that increasing population growth, with its knock-on effects on increased social network connectivity and assortative mixing in urban settings, has played a major role in increasing the transmission rate of HCV in São Paulo and perhaps more widely.

## Supporting Information

Table S1 Bayes factor values obtained for substitution models comparison.
(0.07 MB DOC)

Figure S1 Maximum likelihood phylogenetic trees of HCV subtypes. Phylogenies were inferred using 335 sequences from HCV-1a, 499 sequences from HCV-1b and 252 HCV-3a sequences (i.e. a combination of those viruses sampled here and those collected from GenBank). Sequences sampled in this work are shown in phylogenies by colored branches, which indicate the four distinct localities in the State of São Paulo: Ribeirão Preto (red), São Bernardo do Campo (green), São Paulo (blue) and São José do Rio Preto (yellow). There was an evident lack of geographical structure in the data. Black branches correspond to global reference sequences collated from GenBank. As Brazilian sequences that fell within clusters of non-Brazilian sequences likely signify recent migration they were excluded from the subsequent phylodynamic analyses.
(1.03 MB TIF)

Figure S2 Bayesian skyline plots of the main subtypes found in São Paulo: HCV-1a, HCV-3a and HCV-1b. Skyline plots describe the mean change (bold line) in genetic diversity under both the relaxed uncorrelated molecular clock (A, C, E) and strict molecular clock (B, D and F) with values of the 95% of high posterior density (HPD) shown in each case (light line).
(0.88 MB TIF)

Figure S3 Relationship between population size in São Paulo and viral dynamics. The graph shows the exponential population growth in São Paulo State in the period 1920 to 2005 (left y-axis) in millions of people and the increase of viral growth rate (estimated using BEAST) during the same time period (right y-axis). For comparison, the TMRCA for each subtype was obtained also with Path-O-Gen, using the best ML phylogeny inferred with GARLi. A summary of the change in time of HCV growth rates and population size in São Paulo is shown in the graph inserted in the upper left of the figure. Dots indicate the growth rate values estimated for each subtype (shown with same colors as in Figures 2 and 3) versus the log of the human population size at the time of the most recent common ancestor (TMRCA) of the subtype.
(0.52 MB TIF)

Figure S4 Higher human population growth correlates with higher rates of HCV growth. (A) Census population growth in São Paulo (red dots) and the UK (blue dots) during the 20th Century. The best-fit curve for both populations indicate linear growth in the UK and exponential growth in the State of São Paulo. (B) By partitioning the population of Brazil and the UK into age groups, it appears that most of the growth in Brazil is taking place among the age group that we found to be at a higher risk of infection by HCV-1a and 3a (20–39 years-old). (C) The population growth rate of HCV- 1a and HCV-3a was consistently higher in Brazil than in the UK, suggesting that human population growth accelerates HCV transmission.
(0.62 MB TIF)
