# Supplementary material for: Social Networks Shape the Transmission Dynamics of Hepatitis C Virus
Source: PLoS One. 2010 Jun 23;5(6):e11170. doi: 10.1371/journal.pone.0011170 (PMC2890415; doi:10.1371/journal.pone.0011170)
Supplement: Table S1 — Bayes factor values obtained for substitution models comparison. (0.07 MB DOC) [file pone.0011170.s001.doc]

**Table S1.A**. Bayes factor values obtained for substitution models comparison of HCV-1a

| Trace HCV-1a | **ln P(model|data)** | **S.E.** | **GTR_SRD** | **HKY** | **HKY_SRD** | **GTR** |
| --- | --- | --- | --- | --- | --- | --- |
| **GTR_SRD** | -6582.827 | +/- 9.185 | - | 86.606 | 94.901 | 181.596 |
| **HKY** | -6782.243 | +/- 4.053 | -86.606 | - | 8.295 | 94.99 |
| **HKY_SRD** | -6801.343 | +/- 5.467 | -94.901 | -8.295 | - | 86.695 |
| **GTR** | -7000.967 | +/- 8 | -181.596 | -94.99 | -86.695 | - |

**Table S1.B**. Bayes factor values obtained for substitution models comparison of HCV-1b

| Trace HCV-1b | **ln P(model|data)** | **S.E.** | **GTR_SRD** | **HKY** | **HKY_SRD** | **GTR** |
| --- | --- | --- | --- | --- | --- | --- |
| **GTR_SRD** | -9141.181 | +/- 4.40 | - | 73.677 | 39.283 | 292.626 |
| **HKY** | -9310.829 | +/- 8.8 | -73.677 | - | -34.394 | 218.948 |
| **HKY_SRD** | -9231.633 | +/- 11.2 | -39.283 | 34.394 | - | 253.343 |
| **GTR** | -9814.977 | +/-15.5 | -292.626 | -218.948 | -253.343 | - |

**Table S1.C**. Bayes factor values obtained for substitution models comparison of HCV-3a

| Trace HCV-3a | **ln P(model|data)** | **S.E.** | **GTR_SRD** | **HKY** | **HKY_SRD** | **GTR** |
| --- | --- | --- | --- | --- | --- | --- |
| **GTR_SRD** | -7263.094 | +/- 7.477 | - | 52.864 | -12.581 | 59.462 |
| **HKY** | -7384.816 | +/- 6.708 | -52.864 | - | -65.444 | 6.599 |
| **HKY_SRD** | -7234.125 | +/- 12.994 | 12.581 | 65.444 | - | 72.043 |
| **GTR** | -7400.01 | +/- 3.008 | -59.462 | -6.599 | -72.043 | - |

**Table S1.D**. Bayes factor values obtained for demographic models comparison of HCV-1a

| **Trace** | **ln P(model|data)** | **S.E.** | **HCV-1a Expo** | **HCV-1a Logis** | **HCV-1aCons** |
| --- | --- | --- | --- | --- | --- |
| HCV-1a Expo | *-6,419,052* | *+/- 1,603* | *-* | *-28,708* | *63,426* |
| **HCV-1a Logis** | -6,485,156 | +/- 1,668 | -28,708 | - | 34,717 |
| **HCV-1a Cons** | -6,565,096 | +/- 1,017 | -63,426 | -63,426 | - |

**Table S1.E**. Bayes factor values obtained for demographic models comparison of HCV-1b

| **Trace** | **ln P(model | data)** | **S.E.** | **HCV-1b Expo** | **HCV-1b Logis** | **HCV-1bCons** |
| --- | --- | --- | --- | --- | --- |
| HCV-1bExpo | -8,816,096 | +/- 1,367 | - | -14,077 | - |
| **HCV-1bLogis** | *-8,783,682* | *+/- 1,438* | *14,077* | *-* | *505,425* |
| **HCV-1bCons** | -9,947,465 | +/- 0,959 | -491,347 | -505,425 | 491,347 |

**Table S1.F**. Bayes factor values obtained for demographic models comparison of HCV-3a

| **Trace** | **ln P(model | data)** | **S.E.** | **HCV-3a Expo** | **HCV-3a Logis** | **HCV-3aCons** |
| --- | --- | --- | --- | --- | --- |
| HCV-3a Expo | *-6,946,683* | *+/- 1,554* | *-* | *15,635* | *155,053* |
| **HCV-3a Logis** | -6,982,685 | +/- 1,841 | -15,635 | - | 139,418 |
| **HCV-3a Cons** | -7,303,707 | +/- 1,03 | -155,053 | -139,418 | - |
