# Supplementary figures and images for: Social Networks Shape the Transmission Dynamics of Hepatitis C Virus
Source: PLoS One. 2010 Jun 23;5(6):e11170. doi: 10.1371/journal.pone.0011170 (PMC2890415; doi:10.1371/journal.pone.0011170)

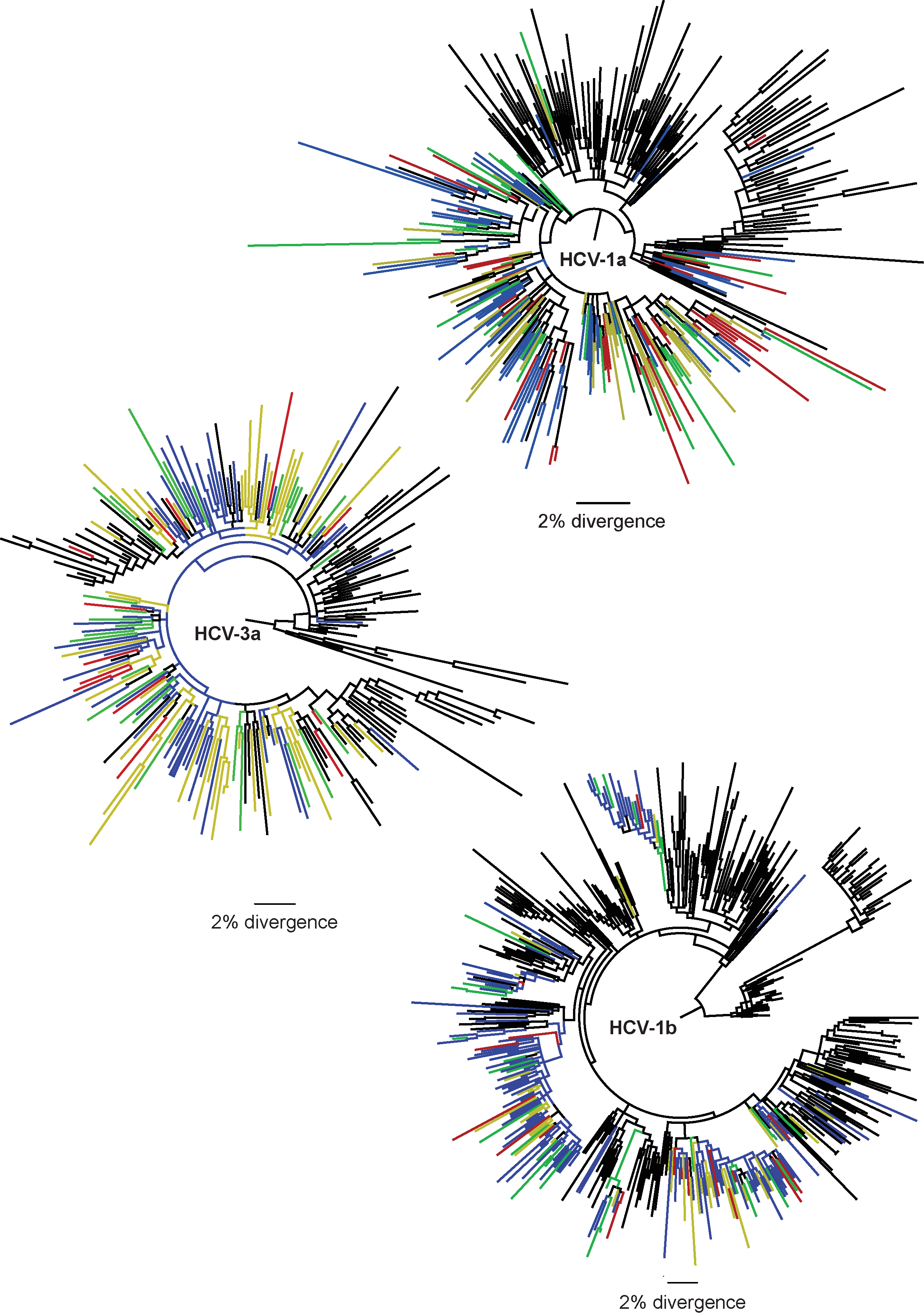

Supplement: Figure S1 — Maximum likelihood phylogenetic trees of HCV subtypes. Phylogenies were inferred using 335 sequences from HCV-1a, 499 sequences from HCV-1b and 252 HCV-3a sequences (i.e. a combination of those viruses sampled here and those collected from GenBank). Sequences sampled in this work are shown in phylogenies by colored branches, which indicate the four distinct localities in the State of São Paulo: Ribeirão Preto (red), São Bernardo do Campo (green), São Paulo (blue) and São José do Rio Preto (yellow). There was an evident lack of geographical structure in the data. Black branches correspond to global reference sequences collated from GenBank. As Brazilian sequences that fell within clusters of non-Brazilian sequences likely signify recent migration they were excluded from the subsequent phylodynamic analyses. (1.03 MB TIF) [file pone.0011170.s002.tif]

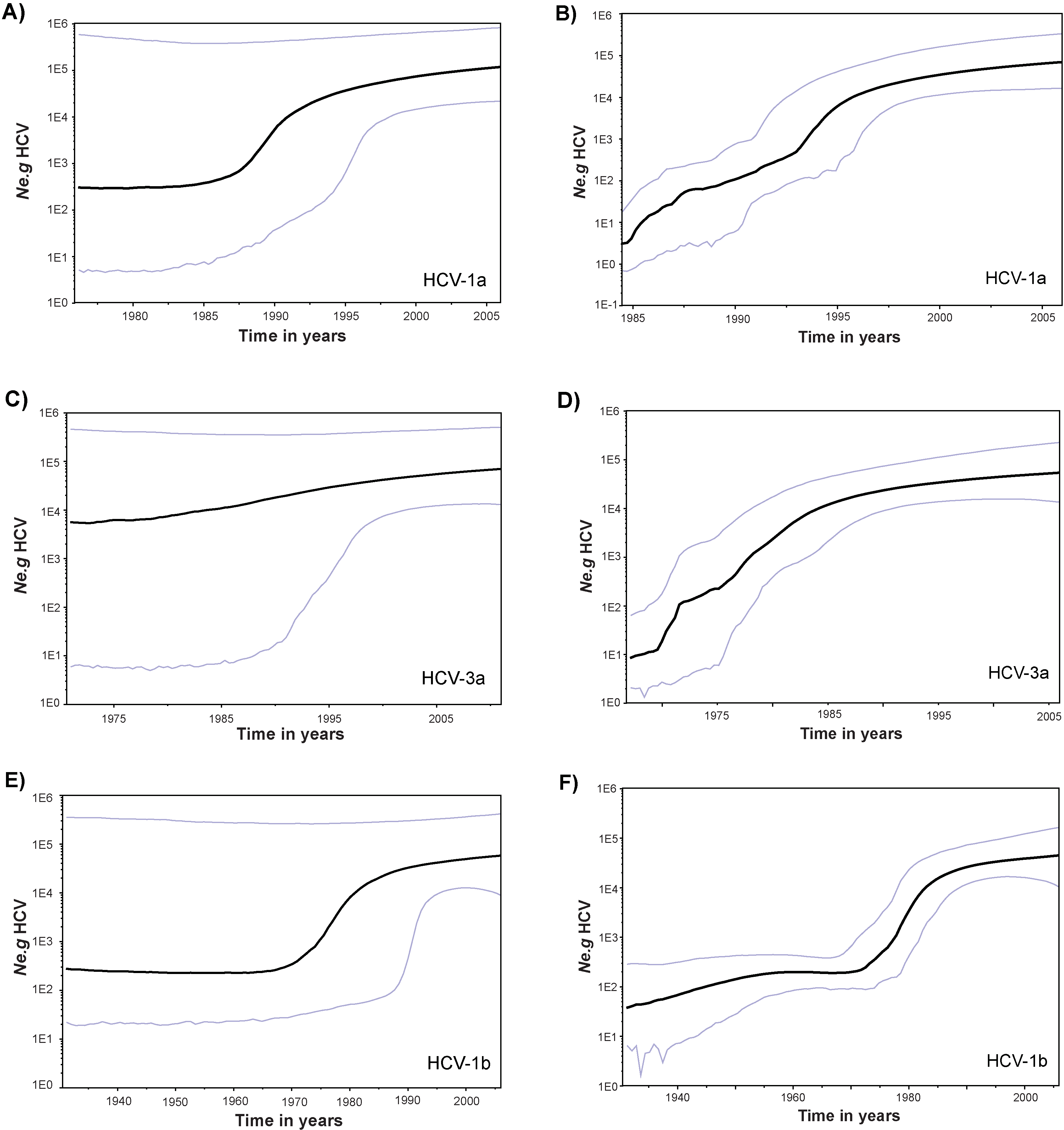

Supplement: Figure S2 — Bayesian skyline plots of the main subtypes found in São Paulo: HCV-1a, HCV-3a and HCV-1b. Skyline plots describe the mean change (bold line) in genetic diversity under both the relaxed uncorrelated molecular clock (A, C, E) and strict molecular clock (B, D and F) with values of the 95% of high posterior density (HPD) shown in each case (light line). (0.88 MB TIF) [file pone.0011170.s003.tif]

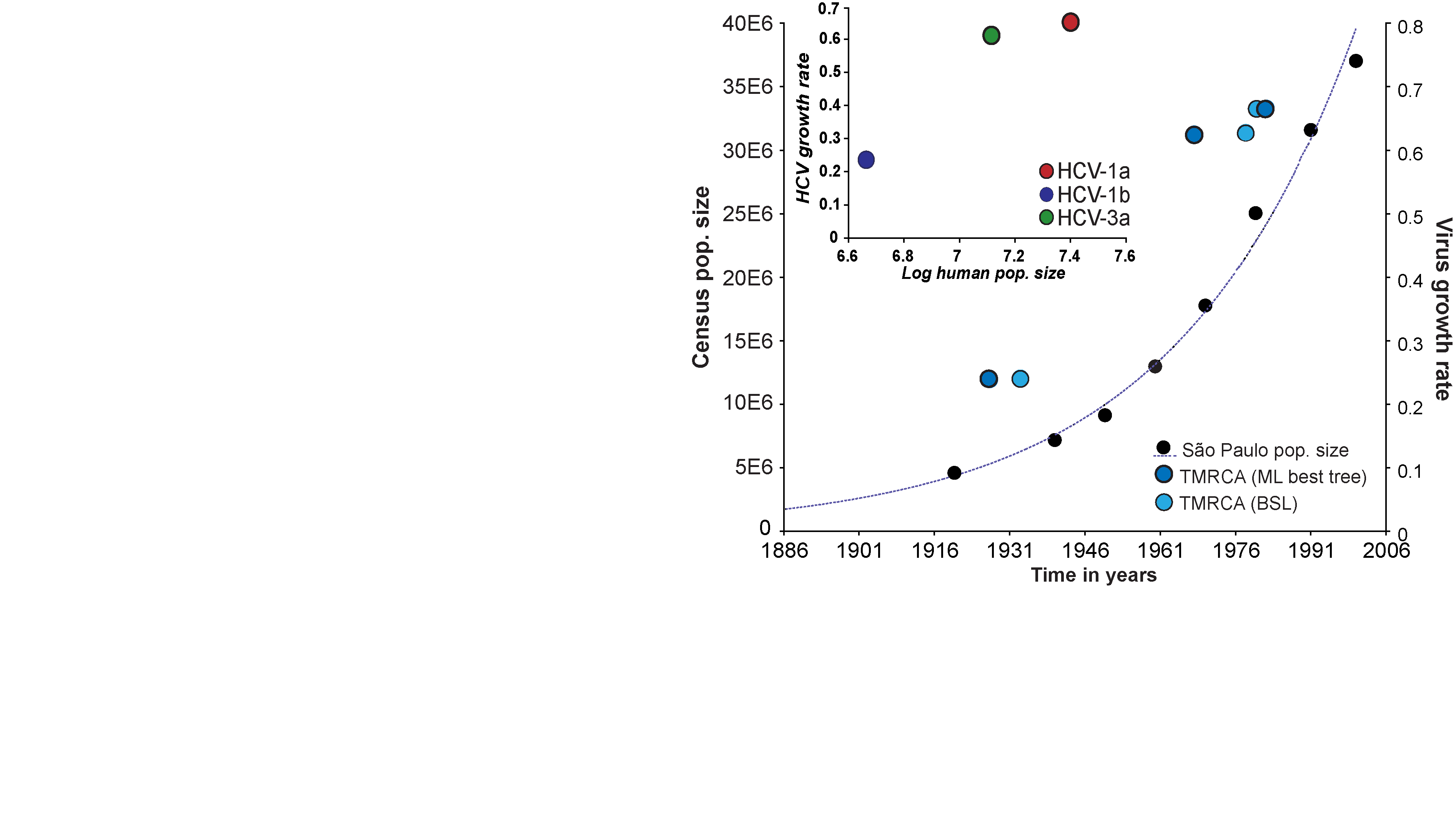

Supplement: Figure S3 — Relationship between population size in São Paulo and viral dynamics. The graph shows the exponential population growth in São Paulo State in the period 1920 to 2005 (left y-axis) in millions of people and the increase of viral growth rate (estimated using BEAST) during the same time period (right y-axis). For comparison, the TMRCA for each subtype was obtained also with Path-O-Gen, using the best ML phylogeny inferred with GARLi. A summary of the change in time of HCV growth rates and population size in São Paulo is shown in the graph inserted in the upper left of the figure. Dots indicate the growth rate values estimated for each subtype (shown with same colors as in Figures 2 and 3) versus the log of the human population size at the time of the most recent common ancestor (TMRCA) of the subtype. (0.52 MB TIF) [file pone.0011170.s004.tif]

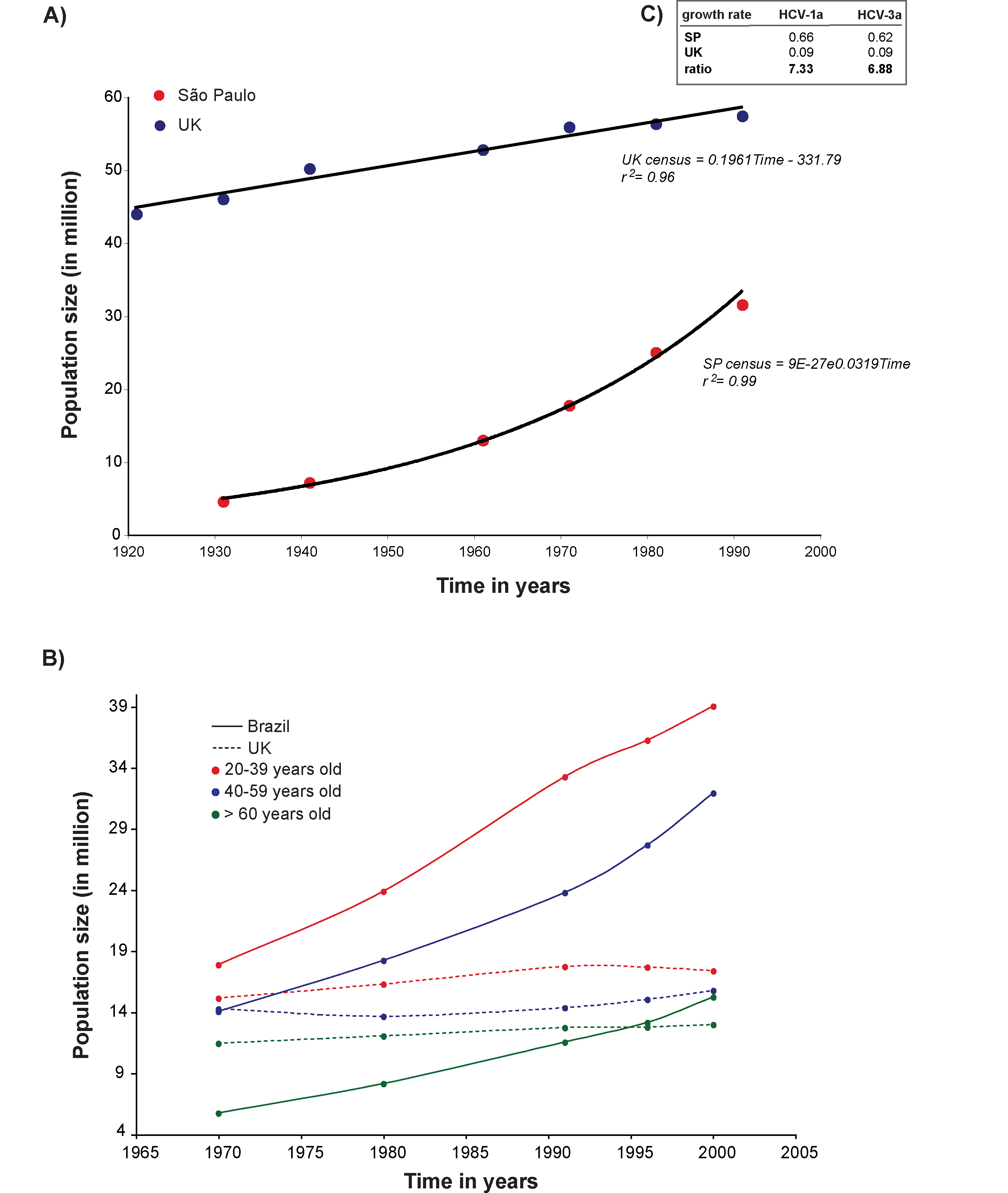

Supplement: Figure S4 — Higher human population growth correlates with higher rates of HCV growth. (A) Census population growth in São Paulo (red dots) and the UK (blue dots) during the 20th Century. The best-fit curve for both populations indicate linear growth in the UK and exponential growth in the State of São Paulo. (B) By partitioning the population of Brazil and the UK into age groups, it appears that most of the growth in Brazil is taking place among the age group that we found to be at a higher risk of infection by HCV-1a and 3a (20–39 years-old). (C) The population growth rate of HCV- 1a and HCV-3a was consistently higher in Brazil than in the UK, suggesting that human population growth accelerates HCV transmission. (0.62 MB TIF) [file pone.0011170.s005.tif]
